# Highly Sensitive SERS Detection of Food Colorants via Charge Transfer of Metal and Semiconductor in Ag/TiO_2_/Ti Foam

**DOI:** 10.3390/foods14233998

**Published:** 2025-11-22

**Authors:** Qunlong Wang, Yuting Jing, De Zhang, Ruijing Wang, Linlin Chen, Jianghua Zhang, Shaofeng Sui, Xuefeng Wang

**Affiliations:** 1 Shanghai Key Lab of Chemical Assessment and Sustainability, School of Chemical Science and Engineering, Tongji University, Shanghai 200092, China; wangql412@163.com (Q.W.); jyt1215@163.com (Y.J.); zinkchang0923@gmail.com (D.Z.); 2231003@tongji.edu.cn (L.C.); 2 Shanghai Municipal Center for Disease Control and Prevention, Shanghai 200336, China; zhangjianghua@scdc.sh.cn (J.Z.); suishaofeng@scdc.sh.cn (S.S.)

**Keywords:** pulsed laser deposition, surface-enhanced Raman scattering, Ag/TiO_2_/Ti foam, charge transfer mechanism

## Abstract

A three-dimensional Ag/TiO_2_/Ti foam was fabricated via thermal annealing followed by pulsed laser deposition (PLD), providing a simple and scalable fabrication strategy. The porous Ti foam framework allows for the uniform dispersion of Ag nanoparticles (NPs), while the thermally formed TiO_2_ interlayer promotes synergistic electromagnetic and chemical enhancement mechanisms. The localized electromagnetic field amplification at Ag-TiO_2_ interfaces was simulated using the finite-difference time-domain (FDTD) method. Density functional theory (DFT) calculations confirmed that TiO_2_ enhances both rhodamine 6G (R6G) adsorption on the substrate and charge transfer (CT) between the substrate and R6G, increasing the SERS activity. The optimized substrate demonstrates exceptional surface-enhanced Raman scattering (SERS) performance with an enhancement factor of 1.9 × 10^7^ and a detection limit of 2.24 × 10^−11^ M for rhodamine 6G, with good reproducibility (RSD = 8.4%). Practical applicability is validated through sensitive detection of food colorants (brilliant blue and allura red). The synergistic combination of CT and electromagnetic enhancement in the easily fabricated Ag/TiO_2_/Ti foam enables its application as a promising platform for food safety monitoring, effectively bridging laboratory innovation and practical applications.

## 1. Introduction

Artificial food colorants, which are widely used to enhance the visual appeal and marketability of food products, have raised growing concerns over public health and food safety worldwide. While these synthetic additives improve the appearance of food, many colorants have been linked to adverse health outcomes, including allergic reactions, neurotoxicity, and an increased risk of cancer [1,2,3]. Due to their potential health risks to humans, the application of artificial food colorants is strictly regulated in many countries [4]. Although conventional detection methods such as high-performance liquid chromatography (HPLC) [5,6] and liquid chromatography–mass spectrometry (LC-MS) [7,8] offer high accuracy, they suffer from several limitations. These include complex and time-consuming sample preparation, reliance on expensive equipment, and the need for specialist operators [9]. These drawbacks make them unsuitable for the on-site, rapid screening of large batches of food products. In recent years, surface-enhanced Raman scattering (SERS) has become the ideal solution for monitoring food colorants thanks to its advantages of ultra-sensitivity, simple operation, and minimal sample consumption.

Since SERS was first observed on roughened silver surfaces in 1970s [10], this technique has been widely applied in diverse fields such as environmental monitoring [11,12], biological and biomedical sciences [13,14], as well as food safety [15,16]. A key advantage of SERS is its exceptional sensitivity attributes that stem from its unique ability to enhance Raman signals by several orders of magnitude. This signal enhancement is primarily driven by two core mechanisms, namely electromagnetic effects and chemical effects, which collectively enable the detection of trace analytes at extremely low concentration levels [17,18]. The versatility and sensitivity of SERS make it a promising tool for addressing complex analytical challenges in both scientific research and practical applications.

The performance of SERS critically depends on the design of active substrates, which directly determine signal enhancement efficiency. Early efforts focused on coinage metals (Ag, Au, Cu) due to their strong plasmonic effects and ability to generate intense electromagnetic fields [19,20]. While coinage metals remain superior in performance, research has extended to semiconductor materials [21,22,23] because of their additional charge transfer (CT) mechanism [24], which demonstrate distinct advantages over noble metals in tunable band gaps, photoluminescence, and environmental stability [25]. For instance, double-shelled hollow ZnO microspheres [23] were constructed to achieve highly selective SERS detection through the synergistic effects of CT resonance and Mie resonance (refers to light scattering by spherical or quasi-spherical particles) [26]. TiO_2_ has garnered significant interest as a semiconductor material due to its non-toxicity, photocatalytic degradability, chemical stability, and biocompatibility [27,28,29]. Liu et al. engineered Yb-doped TiO_2_ to modulate its band structure and electronic properties, thereby enhancing CT efficiency and achieving ultrasensitive SERS detection with a limit of 1 × 10^−9^ M [22]. The chemical enhancement of TiO_2_ via molecule–semiconductor interactions synergizes with the electromagnetic fields of Ag, leading to multiplicative SERS amplification [30]. With Ag-TiO_2_ hybrids, photoexcited electrons from Ag NPs can inject into the conduction band of TiO_2_, creating an additional CT pathway [31].

Despite the promising performance of Ag/TiO_2_ composites, the synthesis methods often involve multi-step chemical reactions [32,33], or toxic reagents [28,34], limiting their practical applicability. In this paper, we propose a facile strategy using pulsed laser deposition (PLD) to fabricate Ag/TiO_2_/Ti foam substrates. This method allows for the uniform distribution of Ag NPs on a porous foam structure, while the TiO_2_ layer is formed through an annealing process. The SERS enhancement mechanism of Ag/TiO_2_/Ti foam was analyzed by finite-difference time-domain (FDTD) simulations and density functional theory (DFT) calculations. It was found that the annealed TiO_2_ layer not only facilitates the adsorption of rhodamine 6G (R6G) on the substrate surface but also enhances CT between the substrate and R6G. Subsequently, the as-prepared Ag/TiO_2_/Ti foam substrates were employed for the detection of target food colorants (brilliant blue and allura red). The experiment proved the great potential of Ag/TiO_2_/Ti foam substrate for food colorant detection. The resulting substrate combines the advantages of high surface area, abundant “hot spots”, and efficient CT, offering a simple, safe, and scalable platform for high-performance food monitoring.

## 2. Experimental Section

### 2.1. Deposition of Ag NPs

**Figure 1:**
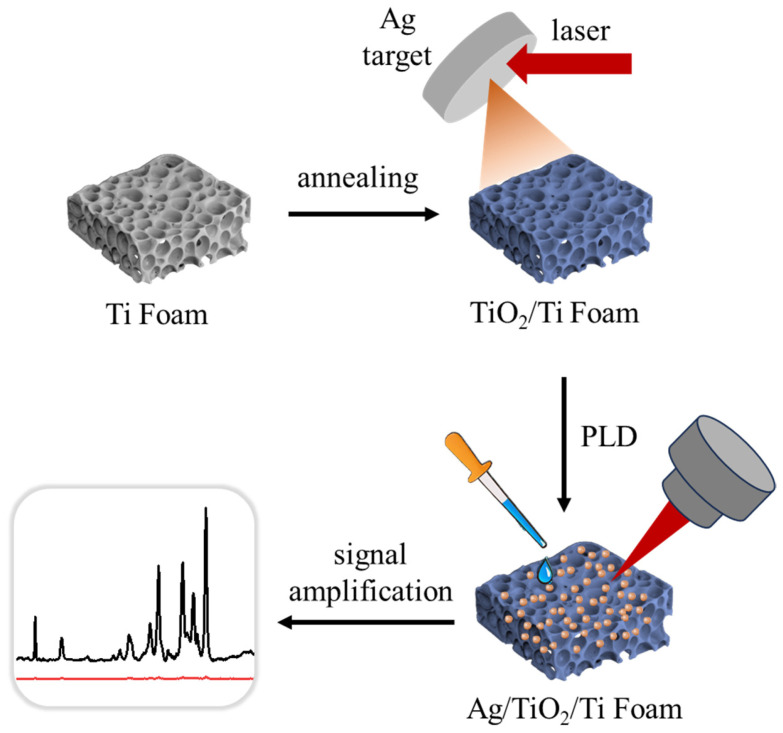
Schematic illustration of the Ag/TiO_2_/Ti foam preparation process.

Figure 1 illustrates the preparation workflow for Ag/TiO_2_/Ti foam. First, Ti foams and Ti foils (Sinopharm Chemical Reagent Co., Ltd. CN) underwent ultrasonic cleaning in acetone, ethanol, and deionized water sequentially, with each step lasting for 10 min. Acetone, ethanol, and DI water were used for cleaning to sequentially remove organic contaminants, residual impurities, and ionic residues from the substrate surface, ensuring a clean and interference-free interface for subsequent experiments. A TiO_2_ layer was then grown on the Ti foam surface via air annealing at 500 °C for 2 h. Ag NPs were deposited using the PLD technique [35,36,37], in which the pulsed laser is focused on the rotating metal Ag target (purity > 99.99%, Alfa Aesar, USA)) to produce the plasma and then it diffuses to the TiO_2_/Ti surface to form the Ag NPs. In detail, the vacuum chamber is first pumped to be below 10^−4^ Pa. Then, an Nd:YAG laser with a wavelength of 1064 nm (8 ns pulse duration, 10 Hz repetition frequency) is focused on an Ag target with a diameter of 12 mm at room temperature. Deposition times (20–40 min) were optimized for SERS performance (Figures S1 and S2), and the optimal time for Ag/TiO_2_/Ti foam was 30 min. For comparative analysis, Ag NPs were deposited on Ti foils following identical parameters.

### 2.2. Characterization

To characterize the samples, field emission scanning electron microscopy (FESEM; Hitachi S4800, Tokyo, Japan) equipped with energy-dispersive spectroscopy (EDS, Tokyo, Japan) was used to record morphologies and elemental distribution. FESEM is a high-resolution imaging technique that generates surface topography images by scanning the sample with a focused electron beam, enabling direct observation of Ag NPs’ dispersion and substrate porous structure. EDS is an elemental analysis technique coupled with SEM, which detects characteristic X-rays emitted from the sample upon electron beam irradiation to quantify elemental composition. It helped confirm the uniform presence of Ag, Ti, and O elements in the composite substrate. The mean size of Ag NPs was determined by analyzing FESEM micrographs using ImageJ software (Version 1.51J) combined with manual counting. Transmission electron microscopy (TEM; JEOL JEM 2100 F, Tokyo, Japan) was employed to examine the microstructure of Ag NPs and the TiO_2_ layer. TEM transmits electrons through thin sample sections to produce high-resolution internal structure images, allowing for the verification of Ag NPs’ crystalline nature. X-ray diffraction (XRD; Bruker Focus D8 with Cu Ka radiation, Germany) was used to analyze the phase structure: XRD measures the diffraction patterns of X-rays scattered by crystalline materials, which can be matched to standard diffraction databases to identify crystalline phases. X-ray Photoelectron Spectroscopy (XPS; Thermo Scientific K-Alpha, USA) was utilized to investigate the chemical state and surface composition: XPS detects photoelectrons emitted from the sample surface upon X-ray irradiation to determine elemental valence states and chemical bonding environments, which is essential for verifying the interfacial interaction between Ag NPs and TiO_2_.

Raman spectroscopy is a vibrational spectroscopic technique that detects inelastic scattering (Raman scattering, Rainshaw Invia, UK) of monochromatic light by molecules, with Raman peaks corresponding to specific molecular vibrational modes, enabling the identification of target analytes. Raman and SERS spectra were acquired using a Renishaw Invia microscopy Raman spectrometer (514.5 nm laser, 2 mW power, 10 s acquisition time, 50× objective). To verify the SERS activity of the substrates, rhodamine 6G (R6G) and food colorants (brilliant blue, allura red; Sinopharm Chemical Reagent Co., Ltd., CN) at different concentrations served as analytes: 20 µL aliquot of the analyte solution was deposited onto the substrate, and SERS tests were conducted only after the solvent had completely evaporated.

## 3. Results and Discussion

### 3.1. Characterization of Ag/TiO_2_/Ti Foam

**Figure 2:**
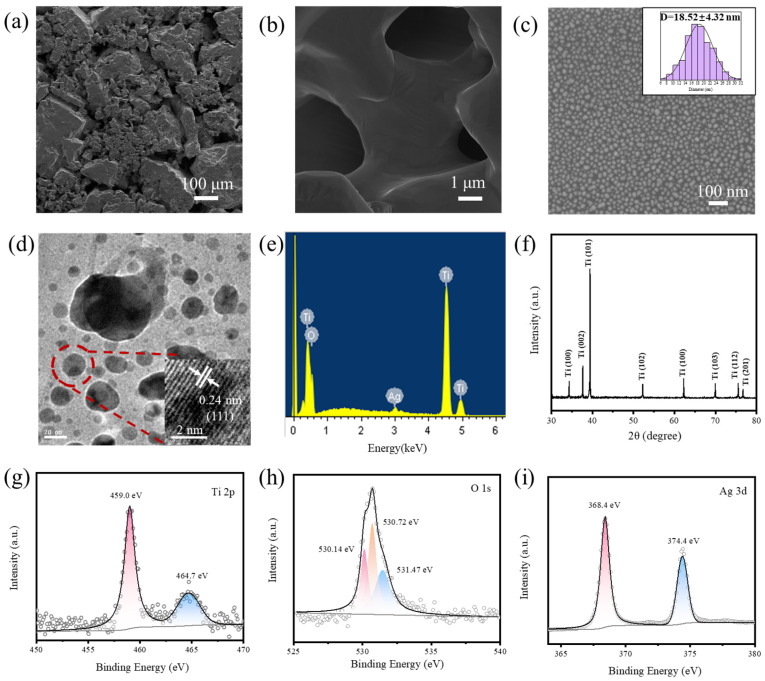
Structural and compositional characterization of Ag/TiO_2_/Ti foam. FESEM images of (**a**) and (**b**) annealed Ti foam and (**c**) Ag/TiO_2_/Ti foam and at different magnifications, inset: the size distribution diagram of Ag NPs. (**d**) TEM image of Ag/TiO_2_/Ti foam, inset: a lattice resolved TEM image of Ag NPs. (**e**) EDS graph of Ag/TiO_2_/Ti foam. (**f**) XRD profile of Ag/TiO_2_/Ti foam. High-resolution XPS spectra corresponding to (**g**) Ti 2p, (**h**) O 1s, and (**i**) Ag 3d.

The morphologies of the annealed Ti foam are presented in Figure 2a,b. As observed, the annealed Ti foam is composed of numerous Ti blocks, each featuring multiple pores. This rough, porous structure provides a large specific surface area, resulting in an abundance of SERS-active sites. Numerous Ag NPs were uniformly distributed across the annealed Ti foam surface, as shown in Figure 2c. The size distribution histogram (inset of Figure 2c) indicates that the NPs within the field of view have diameters ranging from 6 to 32 nm, with an average size of ~18.52 nm for these nearly spherical particles. TEM images of the Ag/TiO_2_/Ti foam (Figure 2d) confirm the random distribution of Ag NPs on the Ti foam, with sizes consistent with those measured from FESEM images. The lattice-resolved TEM image (inset of Figure 2d) demonstrates the high crystallinity of the Ag NPs, where the 0.24 nm crystal planar distance corresponds to the (111) plane of Ag [32]. The EDS graph of Ag/TiO_2_/Ti foam in Figure 2e shows the existence of Ti, O, and Ag elements, indicating the presence of a TiO_2_ layer on the surface of the Ti foam. Elemental composition of the Ag/TiO_2_/Ti from EDS is 16.19, 81.19, and 2.62 of mass% and 37.05, 62.06, and 0.89 atomic% for the O, Ti, and Ag elements, respectively.

XRD was used to examine the phase composition of the annealed Ti foam. Figure 2f reveals that all characteristic diffraction peaks correspond to Ti. These peaks specifically match the (100), (002), (101), (102), (110), (103), (112), and (201) crystal planes of Ti (JCPDS No. 44-1294), with no TiO_2_-related peaks detected. The absence of TiO_2_ diffraction peaks is attributed to the formation of an amorphous oxide layer on the Ti foam surface, as previously reported [38,39]. Meanwhile, the lack of Ag-related diffraction peaks in the XRD pattern is mainly ascribed to the low loading amount and small particle size of Ag NPs on the annealed Ti foam [40].

XPS was used to analyze the elemental composition and valence states of the Ag/TiO_2_/Ti foam, and the results are presented in Figure 2g–i. Figure 2g shows two distinct characteristic peaks for Ti 2p, with the peaks at 459.0 eV and 464.7 eV corresponding to the binding energies of Ti 2p_3/2_ and Ti 2p_1/2_, respectively. The 5.7 eV separation between these two Ti 2p signals matches the value previously reported for TiO_2_ [41], confirming the presence of a TiO_2_ oxide layer on the surface of the annealed Ti foam. For the O 1s spectrum (Figure 2h), deconvolution yielded three peaks at 530.14 eV, 530.72 eV, and 531.47 eV. The peak at 530.14 eV is assigned to Ti–O bonds in TiO_2_, whereas the 531.47 eV peak relates to –OH groups derived from adsorbed H_2_O or oxides at the material surface [42]. The peak at 530.72 eV is likely due to O_2_ adsorption on Ag NPs during exposure to air [36]. Figure 2i shows the characteristic peaks of Ag 3d at 368.4 eV and 374.4 eV, which are attributed to Ag 3d_5/2_ and Ag 3d_3/2_ states, respectively. The energy difference of 6.0 eV between the Ag 3d_5/2_ and Ag 3d_3/2_ binding energies is characteristic of metallic Ag [43], indicating that Ag is deposited on the surface of annealed Ti foam.

### 3.2. SERS of Ag/TiO_2_/Ti Foam

**Figure 3:**
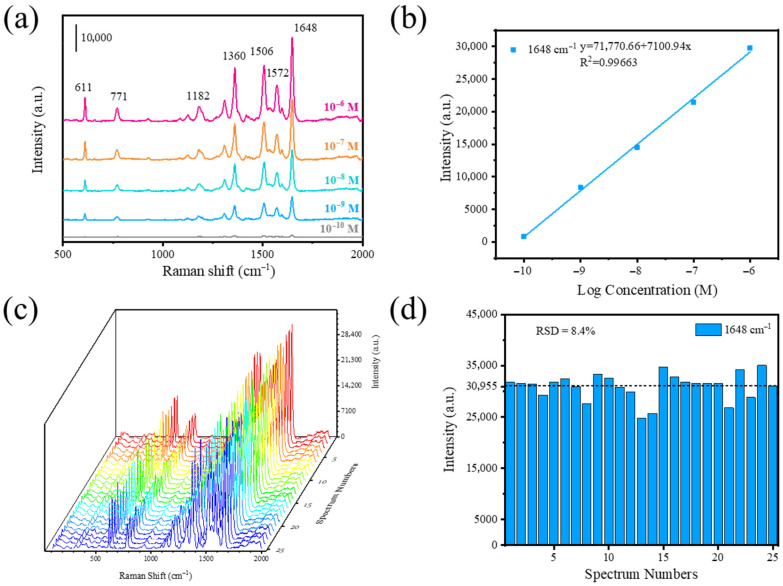
(**a**) SERS spectra of R6G at varying concentrations from 10^−6^ M to 10^−10^ M. (**b**) Linear relationship between the logarithmic SERS intensity (1648 cm^−1^) and the corresponding concentration of R6G. (**c**) SERS spectra acquired from 25 random spots across 5 substrates fabricated under identical conditions, with 5 spots selected randomly from each substrate. (**d**) Histogram of SERS peaks intensity of 10^−6^ M R6G, measured at 25 randomly chosen positions on the Ag/TiO_2_/Ti foams.

SERS performance was verified by detecting different concentrations of R6G using Ag/TiO_2_/Ti foam, as shown in Figure 3a. Peaks at 611 and 771 cm^−1^ originated from C–H out-of-plane bending and C–C–C ring in-plane vibrating, respectively; peaks at 1182 and 1572 cm^−1^ corresponded to C–H in-plane bending and N–H in-plane bending, respectively; and peaks at 1360, 1506, and 1648 cm^−1^ were assigned to aromatic C−C stretching [37]. All peaks are consistent with the characteristic peaks of R6G. Reducing the R6G concentration led to a gradual decrease in SERS signal intensity; however, the Ag/TiO_2_/Ti foam substrate still produced a distinct SERS signal even at a low concentration of 10^−9^ M. When the R6G concentration was reduced to 10^−10^ M, a weak SERS signal was detected at 1648 cm^−1^.

It has been noted that the limit of detection (LOD) is a prominent parameter for SERS performance and is essential for practical application. The logarithmic intensity of 1648 cm^−1^ versus the logarithmic concentration of R6G for Ag/TiO_2_/Ti foam are illustrated in Figure 3b. The linear correlation coefficient R^2^ of R6G was calculated as 0.98215, indicating a positive correlation between the logarithm of SERS intensity and the logarithm of the molecular concentration [44]. The corresponding LODs were calculated as 1.82 × 10^−10^ M based on the three times signal-to-noise ratio estimated from the weak signal of the blank substrates [45]. This confirmed the excellent SERS sensitivity of Ag/TiO_2_/Ti foam for trace and quantitative analysis.

The enhancement factor (EF) is another significant parameter for evaluating SERS performance. The peak intensity of R6G at 1648 cm^−1^ was employed to calculate the EF of Ag/TiO_2_/Ti foam according to the following equation:

$$EF=\frac{I_{SERS}}{I_{NR}}\times \frac{C_{\mathrm{NR}}}{C_{SERS}}$$

where *I_SERS_* denotes the *SERS* intensities of 10^−9^ M R6G on Ag/Ti foam and *I_NR_* refers to the normal Raman scattering intensity of 10^−2^ M R6G on Ti foil (Figure S3), respectively. Similarly, *C_SERS_* and *C_NR_* represent the R6G concentrations on the SERS substrates and bare substrates, respectively. The EF of 1.9 × 10^7^ was obtained, which confirms that the substrate is capable of sensitive SERS detection.

Good SERS signal reproducibility is widely recognized as critical for practical applications. SERS spectra in Figure 3c were acquired from five randomly chosen locations on each of the five substrates from different batches, with all measurements performed under identical experimental conditions. The relative standard deviation (RSD) value of the signal intensities at 1648 cm^−1^ was 8.4%, as shown in Figure 3d. The results show that Ag NPs deposited by the PLD technique have good homogeneity, and the annealed Ti foams provided a good plane for uniform loading of these Ag NPs. Additionally, the Ag/TiO_2_/Ti foam samples stored in air for different durations were characterized, as illustrated in Figure S4. Despite gradual attenuation of SERS signals caused by Ag oxidation, the substrate retained detectable R6G signals for up to 3 months in ambient conditions, demonstrating good stability. Therefore, Ag/TiO_2_/Ti foam with high activity, good reproducibility, and stability will be a candidate for an ideal SERS substrate.

### 3.3. Mechanism

**Figure 4:**
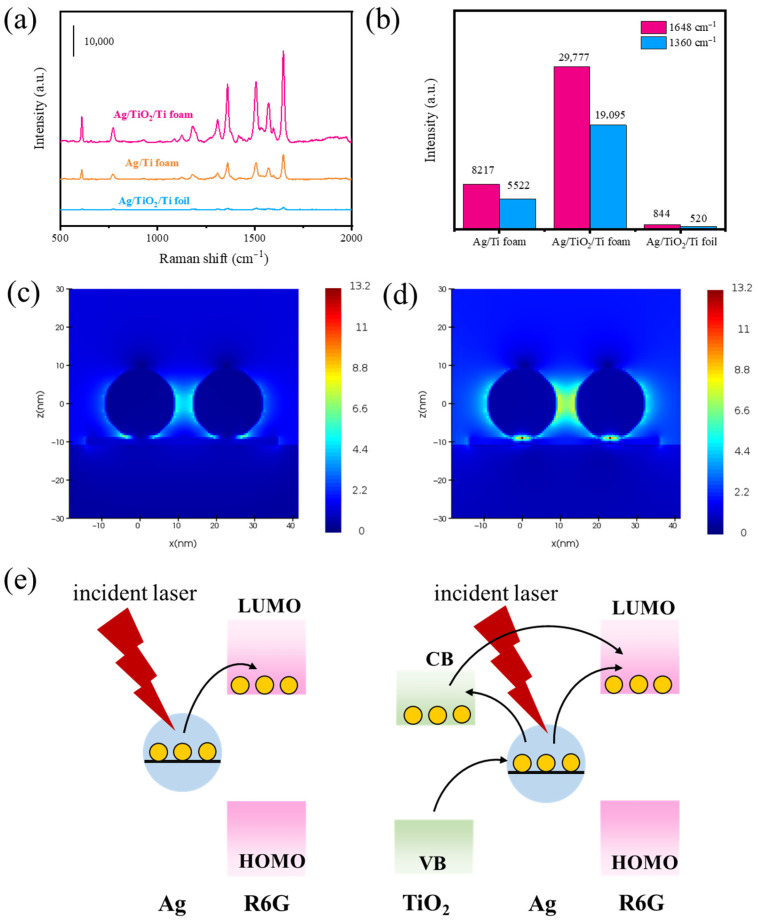
(**a**) SERS spectra of 10^−6^ M of R6G adsorbed on Ag/TiO_2_/Ti foam, Ag/Ti foam, and Ag/TiO_2_/Ti foil. (**b**) The intensity at 1648 and 1360 cm^−1^ towards 10^−6^ M R6G on Ag/TiO_2_/Ti foam, Ag/Ti foam, and Ag/TiO_2_/Ti foil. (**c**) Schematic illustration of CT mechanism in Ag/R6G and Ag/TiO_2_/R6G systems. (**d**,**e**) FDTD-simulated electromagnetic field distributions of Ag NPs on Ti and TiO_2_.

To verify the SERS performance of Ag/TiO_2_/Ti foam, the SERS spectra of R6G adsorbed on Ag/TiO_2_/Ti foam, Ag/Ti foam, and Ag/TiO_2_/Ti foil were compared as shown in Figure 4a,b. The fabrication processes of the Ag/Ti foam and Ag/TiO_2_/Ti foam are similar, with only the annealing step omitted for the former. It is obvious that Ag/TiO_2_/Ti foam exhibits the best SERS performance, which can be attributed to three synergistic factors. First, the 3D porous structure of Ti foam provides a significantly larger specific surface area than Ti foil, enabling higher-density loading of Ag NPs with sub-10 nm interparticle gaps, thereby creating abundant “hot spots”. Second, thermal annealing generated a TiO_2_ interlayer on the Ti foam surface, which intensifies the local electromagnetic fields at the interface between the Ag and TiO_2_ layers. Third, this TiO_2_ interlayer plays a dual role in enhancing SERS performance. On the one hand, as a wide-bandgap semiconductor, TiO_2_ directly participates in CT with adsorbed molecules [46]; on the other hand, it acts as a “bridge” to facilitate CT between adsorbed molecules and Ag NPs via the “donor–bridge–acceptor” CT mode [31]. Collectively, the dual CT contributions of TiO_2_, combined with the electromagnetic enhancement arising from the Ag-TiO_2_ interface, create a synergistic effect that significantly elevates the SERS signal intensity of the Ag/TiO_2_/Ti foam.

It is now widely accepted that the SERS enhancement mechanisms are electromagnetic enhancement, which can provide enhancement factors of more than 10^6^ times, and chemical enhancement, which can provide enhancement factors of 10–100 times [19]. To further analyze the electromagnetic field enhancement of Ag/TiO_2_/Ti substrates, the magnetic field distributions of Ag NPs on TiO_2_ and Ti were simulated by the FDTD. FDTD simulations were performed with FDTD Solutions from Lumerical Solutions, Inc. (Vancouver, BC, Canada). The diameter of the Ag NPs was set to 18 nm and the gap to 5 nm. The fitting material parameters of Ag, Ti, and TiO_2_ were obtained from the material library in the software and the refractive index database [47]. The thickness of the Ti or TiO_2_ layer was set to 2 nm and the wavelength of the excitation light source was 514.5 nm. As shown in Figure 4c,d, a significant enhancement of the EM can be observed at the junction between Ag NPs, which is attributed to the “hot spot” effect. The simulation results further reveal that the electromagnetic field intensity at the interface of Ag and TiO_2_ is much stronger than that at the interface of Ag and Ti. The maximum electromagnetic field intensity at the interface of Ag and Ti was 6.73, while the intensity at the interface of Ag and TiO_2_ was 13.2. Since SERS activity is influenced by electromagnetic field intensity, the SERS signal of Ag/TiO_2_/Ti substrate exceeds that of Ag/Ti substrate, which is consistent with the findings reported in Figure 4a,b. Under identical simulation conditions, the difference in electromagnetic field intensity originates from the contribution of TiO_2_ to electromagnetic enhancement through the generation of additional fields at the interface between Ag and TiO_2_.

Figure 4e is a schematic diagram of the CT enhancement mechanism of R6G on Ag/TiO_2_/Ti foam. In the Ag and R6G system, the energy difference between the lowest unoccupied molecular orbital (LUMO) of R6G and the Fermi level of Ag is lower than the incident laser energy. Therefore, the electrons are excited from the Fermi level of Ag NP to the LUMO level of R6G under 514.5 nm laser irradiation [46,48]. In the case of the Ag/TiO_2_ and R6G system, the presence of TiO_2_ layer expanded the electron transfer pathway. The incident laser energy is insufficient to excite electrons from the valence band (VB) to the conduction band (CB) of TiO_2_ due to the 3.2 eV bandgap. The electrons are excited from the VB to the Fermi level of Ag NP and then injected into the LUMO of R6G [46]. Moreover, the photoexcited electrons from Ag deposited on TiO_2_ are injected into the CB of TiO_2_ and subsequently transfered to the LUMO of R6G adsorbed on TiO_2_ [31]. Obviously, the chemical enhancement in Ag/TiO_2_ and R6G systems must be caused by the synergistic effect of Ag and TiO_2_ in addition to the CT enhancement of TiO_2_ or Ag itself.

### 3.4. DFT Calculations

**Figure 5:**
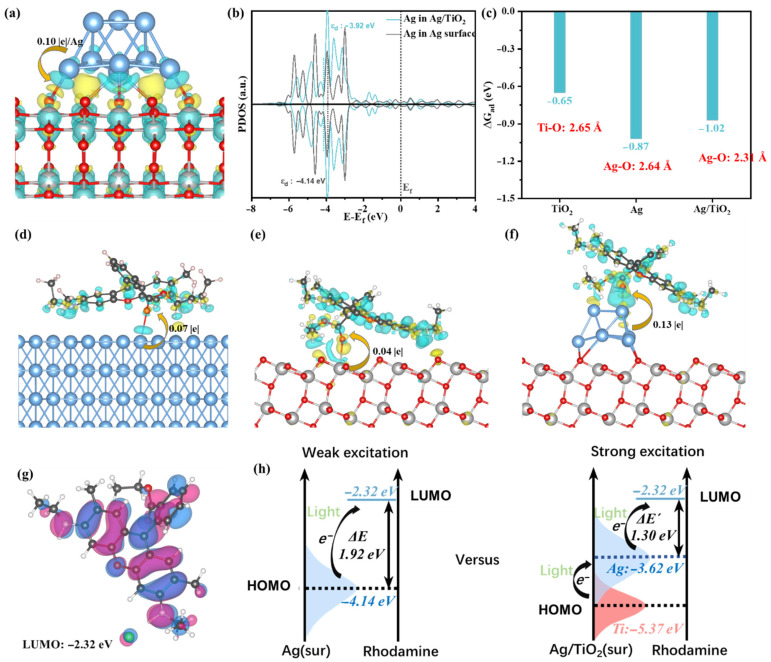
(**a**) The charge density difference diagram of Ag/TiO_2_ model. (**b**) The projected density states (PDOS) of Ag in Ag surface and Ag/TiO_2_ models. (**c**) The adsorption energies (ΔG_ad_) of rhodamine on Ag, TiO_2_, and Ag/TiO_2_ surfaces. (**d**–**f**) The charge density difference diagrams of the adsorption states of rhodamine on Ag, TiO_2_, and Ag/TiO_2_ surfaces, respectively. (**g**) The lowest unoccupied orbital of rhodamine with the orbital energy. (**h**) The electron transfer process from material to rhodamine on Ag and Ag/TiO_2_ surfaces.

The electronic structure of SERS substrates influences the SERS enhancement effect and is closely associated with both electromagnetic and chemical enhancement mechanisms. DFT calculations provide an effective approach to investigate the interfacial charge distribution in composite materials [49]. Consequently, DFT calculations were employed to examine how the electronic structure of Ag/TiO_2_/Ti foam affects its SERS activity. The software and parameters used for the DFT calculations have been listed in the supporting information. Figure 5a shows the charge density difference diagram of the Ag/TiO_2_ model. As evident from this diagram, a distinct charge transfer of 0.1 e per Ag atom occurs from Ag to TiO_2_. The projected density of states (PDOS) of Ag in Ag surface and Ag/TiO_2_ models is shown in Figure 5b. The PDOS of Ag in the Ag/TiO_2_ structure shows multiple intense peaks in the energy range of −2.5 eV to −6 eV. These peaks are associated with the occupied molecular orbitals, suggesting the potential for CT processes. More importantly, the d-band center of Ag atoms shifts from −4.14 eV to −3.92 eV with the effect of TiO_2_, revealing a better electron transfer tendency.

The adsorption energies (ΔG_ad_) of R6G on the surfaces of Ag, TiO_2_, and Ag/TiO_2_ were computed separately, as presented in Figure 5c. The adsorption energy of R6G on Ag/TiO_2_ is −1.02 eV, with a bond length of 2.31 Å for the Ag-O bond. These results indicate that R6G exhibits a greater propensity for adsorption on the Ag/TiO_2_ surface, thereby enhancing its sensitivity to the SERS effect. As observed from the charge density difference diagrams of R6G in its adsorbed states on Ag, TiO_2_, and Ag/TiO_2_ surfaces (Figure 5d–f), the Ag/TiO_2_ surface exhibits the highest electron transfer magnitude. This implies the maximum number of electrons available for excitation, along with the strongest CT effect among the three structures. The excited electrons are primarily transferred to the conjugated ring of the R6G molecule (Figure 5g).

The CT process derived from DFT simulations (Figure 5h) validates the theory presented in Section 3.3. In the system containing only R6G and Ag, electrons transition from the HOMO of Ag to the LUMO of R6G, exhibiting an energy difference of 1.92 eV. In contrast, in the R6G/Ag/TiO_2_ system, electrons first transfer from TiO_2_ to Ag, followed by a transition to the LUMO of R6G, with a corresponding energy difference of 1.30 eV. Thus, the presence of TiO_2_ lowers the barrier for CT and enhances the SERS activity of the substrate.

### 3.5. Applications

**Figure 6:**
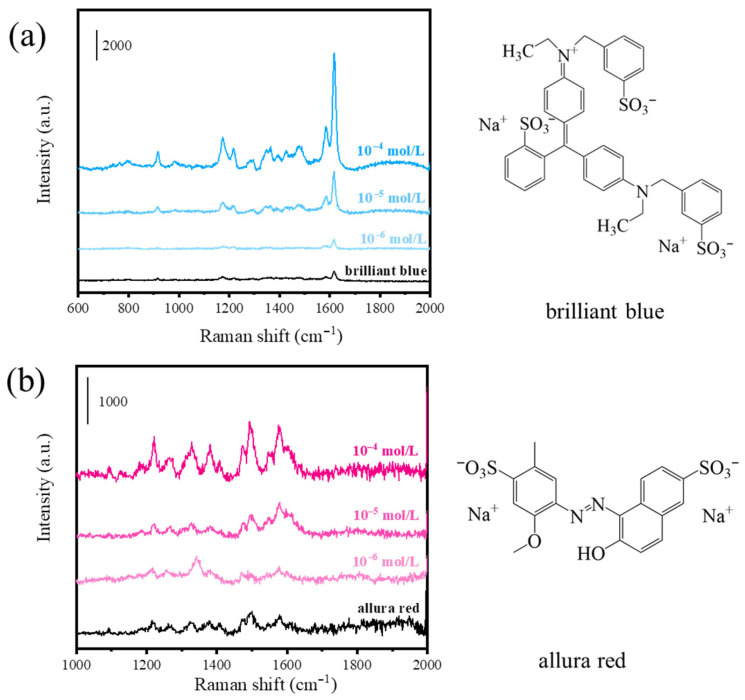
SERS spectra of (**a**) brilliant blue and (**b**) allura red at concentrations ranging from 10^−4^ M to 10^−6^ M. The molecular structures of these food colorants are shown on the right.

The widespread use of artificial food colorants in processed foods has raised concerns regarding their long-term health impacts. Synthetic colorants such as brilliant blue and allura red, valued for their stability and vivid colors, have been associated with neurobehavioral effects and allergic reactions [4]. SERS technology emerges as a robust approach for the rapid and sensitive detection of these colorants. To verify the detection capability of Ag/TiO_2_/Ti foam substrate for food colorants, SERS spectra were recorded for various concentrations of brilliant blue and allura red, as shown in Figure 6. In Figure 6a, multiple distinct characteristic peaks of brilliant blue were observed. The most intense peak, located at 1617 cm^−1^, is associated with aromatic C-C stretching vibrations. Additional characteristic peaks were identified at 1584 cm^−1^, 1218 cm^−1^, 1174 cm^−1^, and 916 cm^−1^, corresponding to N–H in-plane bending vibration, C-H rocking vibration, C-H in-plane bending vibration, and C-H in-plane swing vibration, respectively [50]. Furthermore, the SERS spectra of brilliant blue across varying concentrations exhibited close alignment with the Raman spectrum of its solid powder, achieving a detection limit of 10^−6^ M. These results confirm the feasibility of the substrate for quantitative analysis of brilliant blue.

Figure 6b provides SERS spectroscopy data for allura red, where characteristic peaks were detected at 1221 cm^−1^, 1265 cm^−1^, 1380 cm^−1^, 1496 cm^−1^, and 1578 cm^−1^. These peaks correspond to C-H rocking vibration, C-O stretching vibration, C-H wagging vibration, C-H wagging vibration, and asymmetric stretching vibration of the benzene ring, respectively [51]. The characteristic SERS peaks of allura red are clearly detectable at concentrations as low as 10^−6^ M, showing spectral features generally consistent with those of its powder reference samples. The minor wavenumber shifts observed for allura red adsorbed on Ag/TiO_2_/Ti foam could be associated with molecular orientation effects.

Collectively, the characteristic Raman peaks of both food colorants matched vibrational modes of their functional groups. The Ag/TiO_2_/Ti foam substrate demonstrated the ability to detect both colorants, with the lowest detectable concentration reaching 10^−6^ M. These findings indicate that the Ag/TiO_2_/Ti foam substrate holds promise for trace-level analysis of diverse food colorants.

## 4. Conclusions

In this study, a three-dimensional Ag/TiO_2_/Ti foam substrate was developed via a scalable two-step strategy combining thermal annealing and PLD, demonstrating exceptional performance as a hybrid SERS platform. The substrate achieved an enhancement factor of 1.9 × 10^7^, a detection limit of 2.24 × 10^−11^ M for R6G, and reproducible performance with an RSD of 8.4%. The porous Ti foam framework enabled uniform dispersion of Ag NPs with sub-10 nm gaps, creating high-density hot spots. The thermally grown TiO_2_ layer was found to promote CT at the Ag NP–molecule interface, contributing to chemical enhancement. FDTD simulations quantitatively confirmed that the presence of the TiO_2_ layer contributes additional electromagnetic field enhancement, demonstrating the synergistic improvement in SERS performance through TiO_2_ and Ag. DFT calculations verified that TiO_2_ facilitated CT between Ag and R6G, which reduced the energy barrier from 1.92 eV to 1.30 eV, and enhanced R6G adsorption on the Ag/TiO_2_ surface, with an adsorption energy of −1.02 eV. The successful detection of brilliant blue and allura red at concentrations as low as 10^−6^ M demonstrated the potential of Ag/TiO_2_/Ti foam for practical food safety monitoring. This work establishes a scalable fabrication strategy for high-performance SERS substrates, offering a viable pathway from laboratory development to real-world food quality control applications.

## Data Availability

The original contributions presented in this study are included in the article/Supplementary Materials. Further inquiries can be directed to the corresponding authors.

## Supplementary Materials

The following supporting information can be downloaded at: https://www.mdpi.com/article/10.3390/foods14233998/s1, Figure S1. FESEM images of the Ag/TiO_2_/Ti foam at different Ag deposition time: (a) 10 min, (b) 20 min, (c) 30 min, (d) 40 min. Figure S2. (a) SERS spectra of different Ag deposition time of 10^−6^ M R6G adsorbed on Ag/TiO_2_/Ti foams. (b) Corresponding line graphs of SERS intensities of 1648 and 1360 cm^−1^ from 10^−6^ M R6G versus the Ag deposition time. Figure S3. Raman spectra of 10^−2^ M of R6G adsorbed on Ti foil. Figure S4. (a)The SERS spectra of 10-6 M R6G adsorbed on Ag/TiO_2_/Ti foam stored in air for different days. (b) The relationship between storage time of Ag/TiO_2_/Ti foam and SERS intensity of R6G at 1648 cm^−1^ and 1360 cm^−1^. Figure S5. Optimized geometry of R6G on Ag/TiO_2_ and TiO_2_. Figure S6. Optimized geometry of R6G on Ag. Table S1. Elemental composition as obtained from EDS data. References [52,53,54,55,56,57] are cited in Supplementary Materials.

## References

[1] Leleu C., Boulitrop C., Bel B., Jeudy G., Vabres P., Collet E. (2013). Quinoline Yellow dye-induced fixed food-and-drug eruption. Contact Dermat..

[2] McCann D., Barrett A., Cooper A., Crumpler D., Dalen L., Grimshaw K., Kitchin E., Lok K., Porteous L., Prince E. (2007). Food additives and hyperactive behaviour in 3-year-old and 8/9-year-old children in the community: A randomised, double-blinded, placebo-controlled trial. Lancet.

[3] Feng J., Cerniglia C.E., Chen H. (2012). Toxicological significance of azo dye metabolism by human intestinal microbiota. Front. Biosci.-Elite.

[4] Amchova P., Kotolova H., Ruda-Kucerova J. (2015). Health safety issues of synthetic food colorants. Regul. Toxicol. Pharmacol..

[5] Shi J., Huang M., Yang Q., Xu Y., Wu J., Liu H., Zhang J., Zheng F., Dong W. (2025). Relatively reliable and rapid identification of colorant compounds in food matrices by HPLC-DAD-QTOF-MS combined with theoretical calculation. Food Chem..

[6] Deolindo C.T.P., Hoff R.B., Costa A.C.O. (2025). Development and validation of an HPLC-DAD method for the determination of artificial colorants in açaí pulp and commercial products. Food Res. Int..

[7] Ragab M., Khaled O., Elgendy N., Eissa F., Medhat M. (2025). High-throughput LC-MS/MS method for the determination of 14 illegal dyes in complex food matrices and its application in a large-scale survey in Egypt. J. Hazard. Mater..

[8] Long P., Li Y., Han Z., Zhu M., Zhai X., Jiang Z., Wen M., Ho C.-T., Zhang L. (2024). Discovery of color compounds: Integrated multispectral omics on exploring critical colorant compounds of black tea infusion. Food Chem..

[9] Zhao X., Wang Y., Yao Y., Chen L., Lin B., Zheng W., Zeng Y., Li L., She Y., Guo L. (2023). One-Step, On-Site Chemical Printing of a 3D Plasmon-Coupled Silver Nanocoral Substrate toward SERS-Based POCT. Anal. Chem..

[10] Fleischmann M., Hendra P.J., McQuillan A. (1974). Raman spectra of pyridine adsorbed at a silver electrode. Chem. Phys. Lett..

[11] Yu J., Yang M., Li Z., Liu C., Wei Y., Zhang C., Man B., Lei F. (2020). Hierarchical Particle-In-Quasicavity Architecture for Ultratrace In Situ Raman Sensing and Its Application in Real-Time Monitoring of Toxic Pollutants. Anal. Chem..

[12] Xie Y., Dong X., Cai N., Yang F., Yao W., Huang L. (2023). Application of a Novel Au@ZIF-8 Composite in the Detection of Bisphenol A by Surface-Enhanced Raman Spectroscopy. Foods.

[13] Li H., Zhang K.-L., Kou Y., Xu S., Guo X.-M., Fu S.-Y., Li Z., Zhang Y.-J., Chen X., Li J.-F. (2025). Resonance SERS probe based on the bifunctional molecule IR808 combined with SA test strips for highly sensitive detection of monkeypox virus. Spectrochim. Acta A.

[14] Zhang Y., Wang C., He M., Dai Y., Jiang T. (2025). UCL-SERS dual-mode sensing of NaYF4: Yb3+, Er3+/NaYF4: Yb3+@ Au composite nanoparticles. J. Alloys Compd..

[15] Nilghaz A., Mahdi Mousavi S., Amiri A., Tian J., Cao R., Wang X. (2022). Surface-Enhanced Raman Spectroscopy Substrates for Food Safety and Quality Analysis. J. Agric. Food Chem..

[16] Zhai W., Cao M., Xiao Z., Li D., Wang M. (2022). Rapid Detection of Malathion, Phoxim and Thiram on Orange Surfaces Using Ag Nanoparticle Modified PDMS as Surface-Enhanced Raman Spectroscopy Substrate. Foods.

[17] Ding S.-Y., You E.-M., Tian Z.-Q., Moskovits M. (2017). Electromagnetic theories of surface-enhanced Raman spectroscopy. Chem. Soc. Rev..

[18] Yang B., Chen G., Ghafoor A., Zhang Y.-F., Zhang X.-B., Li H., Dong X.-R., Wang R.-P., Zhang Y., Zhang Y. (2023). Chemical Enhancement and Quenching in Single-Molecule Tip-Enhanced Raman Spectroscopy. Angew. Chem. Int. Ed..

[19] Jing Y., Wang R., Wang Q., Xiang Z., Li Z., Gu H., Wang X. (2021). An overview of surface-enhanced Raman scattering substrates by pulsed laser deposition technique: Fundamentals and applications. Adv. Compos. Hybrid Mater..

[20] Atta S., Watcharawittayakul T., Vo-Dinh T. (2022). Ultra-high SERS detection of consumable coloring agents using plasmonic gold nanostars with high aspect-ratio spikes. Analyst.

[21] Yang L., Qin X., Jiang X., Gong M., Yin D., Zhang Y., Zhao B. (2015). SERS investigation of ciprofloxacin drug molecules on TiO_2_ nanoparticles. Phys. Chem. Chem. Phys..

[22] Liu W., He X., Wang Z., Yuan M., Zhao Z., Ye X., Shang S., Song Z., Huang L., Liu Y. (2024). Geometric and Electronic Structure Modulation to Optimize the Charge Transfer of TiO_2_ for Ultrasensitive and Stable SERS Sensing. Inorg. Chem..

[23] Liu Y., Dang A., Liu X., Han Y., Chen J., Zada A., Sun Y., Yuan Z., Luo F., Li T. (2024). Synergistic Resonances and Charge Transfer in Double-Shelled ZnO Hollow Microspheres for High-Performance Semiconductor-Based SERS Substrates. ACS Appl. Nano Mater..

[24] Chowdhury J. (2015). How the Charge Transfer (CT) Contributions Influence the SERS Spectra of Molecules? A Retrospective from the View of Albrecht’s “A” and Herzberg-Teller Contributions. Appl. Spectrosc. Rev..

[25] Han X.X., Ji W., Zhao B., Ozaki Y. (2017). Semiconductor-enhanced Raman scattering: Active nanomaterials and applications. Nanoscale.

[26] Mie G. (1908). Beiträge zur Optik trüber Medien, speziell kolloidaler Metallösungen. Ann. Phys..

[27] Dong Y., Xie Q., Wu S., Li J., Sun L., Ji W. (2023). Revealing Adsorption Mechanism of p-Mercaptobenzoic Acid with TiO_2_ Surfaces Using Electric Field-Enhanced Semiconductor SERS. J. Phys. Chem. C.

[28] Yang W., Tang J., Ou Q., Yan X., Liu L., Liu Y. (2021). Recyclable Ag-Deposited TiO2 SERS Substrate for Ultrasensitive Malachite Green Detection. ACS Omega.

[29] Xue X., Zhao C., Qiao Y., Wang P., Wang J., Shi J., Liu B., Wang Z., Hou E., Chang L. (2024). A novel three-dimensional porous Ag/TiO_2_ hybrid aerogels with high dense hot spot as effective SERS substrate for ultrasensitive detection. Spectrochim. Acta A.

[30] Huang Y., Zhang S., Jiang S., Xu J. (2024). Improved SERS Performance on Ag-Coated Amorphous TiO_2_ Random Nanocavities by the Enhanced Light–Matter Coupling Effect. ACS Sustain. Chem. Eng..

[31] Yang L., Sang Q., Du J., Yang M., Li X., Shen Y., Han X., Jiang X., Zhao B. (2018). A Ag synchronously deposited and doped TiO_2_ hybrid as an ultrasensitive SERS substrate: A multifunctional platform for SERS detection and photocatalytic degradation. Phys. Chem. Chem. Phys..

[32] Yang J., Song G., Zhou L., Wang X., You L., Li J. (2021). Highly sensitively detecting tetramethylthiuram disulfide based on synergistic contribution of metal and semiconductor in stable Ag/TiO_2_ core-shell SERS substrates. Appl. Surf. Sci..

[33] Huang S., Wu C., Wang Y., Yang X., Yuan R., Chai Y. (2021). Ag/TiO_2_ nanocomposites as a novel SERS substrate for construction of sensitive biosensor. Sensor. Actuat. B-Chem..

[34] Wang Y., Ma S., Yu H., Liu Y., Gao J., Yang L., Zhang M., He G., Sun Z. (2021). Effect of TiO_2_ arrays on surface enhanced Raman scattering (SERS) performance for Ag/TiO_2_ substrates. Nanotechnology.

[35] Jing Y., Wang H., Chen X., Wang X., Wei H., Guo Z. (2014). Pulsed laser deposited Ag nanoparticles on nickel hydroxide nanosheet arrays for highly sensitive surface-enhanced Raman scattering spectroscopy. Appl. Surf. Sci..

[36] Jing Y., Wang H., Zhao J., Yi H., Wang X. (2015). Pulsed laser deposition of Ag nanoparticles on titanium hydroxide/oxide nanobelt arrays for highly sensitive surface-enhanced Raman spectroscopy. Appl. Surf. Sci..

[37] Wang Q., Zheng H., Jing Y., Wang R., Chen L., Zhang J., Sui S., Wang X. (2024). Rapid detection of artificial food colorants with surface enhanced Raman spectroscopy: Engineering a novel gold-induced silver triangle nanosheet. J. Alloys Compd..

[38] Li Z., Wang X., Wang X., Xiao T., Zhang L., Lv P., Zhao J. (2018). Preparation and properties of MnO_2_–TiO_2_ nanotube array composite electrodes using titanium foam as the current collector. Int. J. Hydrogen Energy.

[39] Wang Q., Qiu L., Jia Y., Chang Y., Tan X., Yang L., Chen H. (2019). Design of carbon loaded porous TiO_2_ foams by the hydrothermal-assisted annealing carbonization of fruit residue for solar-driven water evaporation. Sol. Energy Mater. Sol. Cells.

[40] Wang R., Zhang D., Luo S., Jiang L., Wang Q., Chen L., Wei G.-F., Wang X. (2024). The engineered interfacial Pd–O–Ti sites on the TiO2 nanobelts to accelerate water dissociation for the alkaline hydrogen evolution. Electrochim. Acta.

[41] Erdem B., Hunsicker R.A., Simmons G.W., Sudol E.D., Dimonie V.L., El-Aasser M.S. (2001). XPS and FTIR Surface Characterization of TiO2 Particles Used in Polymer Encapsulation. Langmuir.

[42] Wang Q., Qiu L., Tan X., Liu Z., Gao S., Wang R. (2019). Amorphous TiO2 granular nanodisks on porous Ti foam for highly effective solar cells and photocatalysts. J. Taiwan Inst. Chem. Eng..

[43] Han D., Guo B., Li Y., Feng W., Liu K., Wu T., Wan Y., Wang L., Gao M., Liu Y. (2024). Simultaneous photocatalytic degradation and SERS detection of tetracycline with self-sustainable and recyclable ternary PI/TiO_2_/Ag flexible microfibers. Microsyst. Nanoeng..

[44] Liu W., Dong J., Ren Y., Zhou W., Han Q., Zhang C., Ren K., Wang Y., Gao W., Qi J. (2025). Fabrication of plasmonic Au-Ag alloy nanostars for ultrasensitive SERS detection. Spectrochim. Acta A.

[45] Shrivastava A., Gupta V. (2011). Methods for the determination of limit of detection and limit of quantitation of the analytical methods. Chron. Young Sci..

[46] Wang Y., Liu J., Ozaki Y., Xu Z., Zhao B. (2019). Effect of TiO_2_ on Altering Direction of Interfacial Charge Transfer in a TiO_2_-Ag-MPY-FePc System by SERS. Angew. Chem. Int. Ed..

[47] Polyanskiy M.N. (2024). Refractiveindex.info database of optical constants. Sci. Data.

[48] Lombardi J.R., Birke R.L. (2014). Theory of Surface-Enhanced Raman Scattering in Semiconductors. J. Phys. Chem. C.

[49] Farrokhpour H., Ghandehari M. (2019). Theoretical Spectroscopic Study on the Au, Ag, Au/Ag, and Ag/Au Nanosurfaces and Their Cytosine/Nanosurface Complexes: UV, IR, and Charge-Transfer SERS Spectra. J. Phys. Chem. C.

[50] Wu W., Liu L., Dai Z., Liu J., Yang S., Zhou L., Xiao X., Jiang C., Roy V.A.L. (2015). Low-Cost, Disposable, Flexible and Highly Reproducible Screen Printed SERS Substrates for the Detection of Various Chemicals. Sci. Rep..

[51] Yao Y., Wang W., Tian K., Ingram W.M., Cheng J., Qu L., Li H., Han C. (2018). Highly reproducible and sensitive silver nanorod array for the rapid detection of Allura Red in candy. Spectrochim. Acta A.

[52] Kresse G., Hafner J. (1993). Ab initio molecular dynamics for liquid metals. Phys. Rev. B.

[53] Kresse G., Hafner J. (1994). Ab initio molecular-dynamics simulation of the liquid-metal--amorphous-semiconductor transition in germanium. Phys. Rev. B.

[54] Blöchl P.E. (1994). Projector augmented-wave method. Phys. Rev. B.

[55] Kresse G., Joubert D. (1999). From ultrasoft pseudopotentials to the projector augmented-wave method. Phys. Rev. B.

[56] Perdew J.P., Burke K., Ernzerhof M. (1996). Generalized Gradient Approximation Made Simple. Phys. Rev. Lett..

[57] Monkhorst H.J., Pack J.D. (1976). Special points for Brillouin-zone integrations. Phys. Rev. B.

