# Supplementary material for: Highly Sensitive SERS Detection of Food Colorants via Charge Transfer of Metal and Semiconductor in Ag/TiO2/Ti Foam
Source: Foods. 2025 Nov 22;14(23):3998. doi: 10.3390/foods14233998 (PMC12691872; doi:10.3390/foods14233998)
Supplement: Supplementary file 1 [file foods-14-03998-s001.zip › foods-3961151-supplementary.pdf]

Supporting Information

Highly Sensitive SERS Detection of Food Colorants via Charge Transfer of Metal and Semiconductor in Ag/TiO_2/Ti Foam

Qunlong Wang [1], Yuting Jing [1], De Zhang [1], Ruijing Wang [1,*], Linlin Chen [1], Jianghua Zhang [2], Shaofeng Sui [2] and Xuefeng Wang [1,*]

[1] Shanghai Key Lab of Chemical Assessment and Sustainability, School of Chemical Science and Engineering, Tongji University, Shanghai 200092, China; wangql412@163.com (Q.W.); jyt1215@163.com (Y.J.); zinkchang0923@gmail.com (D.Z.); 2231003@tongji.edu.cn (L.C.)

[2] Shanghai Municipal Center for Disease Control and Prevention, Shanghai 200336, China; zhangjianghua@scdc.sh.cn (J.Z.); suishaofeng@scdc.sh.cn (S.S.)

* Correspondence: 18ruijing@tongji.edu.cn (R.W.); xfwang@tongji.edu.cn (X.W.)

Experiment

1 Material

Ti foams and Ti foils were purchased from Kunshan Guangjiayuan New Materials Co., Ltd and Qingyuan Metal Material Co., Ltd, respectively. Ag target (purity > 99.99%) was purchased from Alfa Aesar. Meanwhile, analytical-grade reagents including ethanol (C_2H_5OH), acetone (CH_3COCH_3), rhodamine 6G (R6G), brilliant blue ($C_{37}H_{34}N_2Na_2O_9S_3$), and allura red ($C_{18}H_{14}N_2Na_2O_8S_2$) were acquired from Sinopharm Chemical Reagent Co., Ltd. (Shanghai, China).

2 Calculation Method

In this work, spin-polarized density functional theory (DFT) calculations were conducted using the Vienna Ab-inito Simulation Package (VASP) [1, 2]—a software suite that integrates Projected Augmented Wave (PAW) pseudopotentials [3]. To account for the exchange-correlation effects of energy, the generalized gradient approximation (GGA) approach was employed, with the Perdew-Burke-Ernzerhof (PBE) functional selected [4, 5]. The energy cutoff for plane wave expansions was set to 450 eV. The TiO_2 model was derived from an anatase TiO_2 bulk, with a 101 plane used to cleave a structure containing 4 layers of Ti-O atoms; the Ag model, meanwhile, was obtained by cleaving an Ag bulk along the 111 plane to yield 4 layers of Ag atoms. The Ag/TiO_2 model was built by embedding Ag nanoparticle (9 Ag atoms) on TiO_2 surface. For sampling the first Brillouin zone [6], the Monkhorst-Pack scheme was applied with a k-point separation of 0.05 $Å^{-1}$, and the k-point grid was set to 2×2×1. A vacuum space of 15 Å was introduced above the molecular structure to eliminate

periodic interactions throughout the calculations.

During all calculations, the bottom 2 metal layers were fixed, while all other atoms were allowed to fully relax. Geometry relaxation was performed using the Quasi-Newton method, and the relaxation process was halted when the maximum energy and force on each degree of freedom dropped below 1.0×10^{-5} eV and 0.01 eV Å^{-1}, respectively.

To derive the free energy profile, Gibbs free energies for all the states were computed using the ZPE-corrected DFT total energy, where this corrected energy was taken as the enthalpy at 0 K. The calculation can be performed with:

$$\Delta G = \Delta H - T\Delta S = \Delta E_{DFT} + \Delta E_{ZPE} + \int_{0}^{298.15K} \Delta C_V dT - T\Delta S$$

where ΔE_{DFT} is the total energy difference getting from DFT optimization, ΔE_{ZPE} is the zero-point vibrational energy difference, ΔC_V is the heat capacity difference, T is the kelvin temperature, and ΔS is the entropy difference. The free energy of the gas-phase molecule was calculated using thermodynamic principles, with standard thermodynamic data from the standard state employed (ref to www.nist.com).

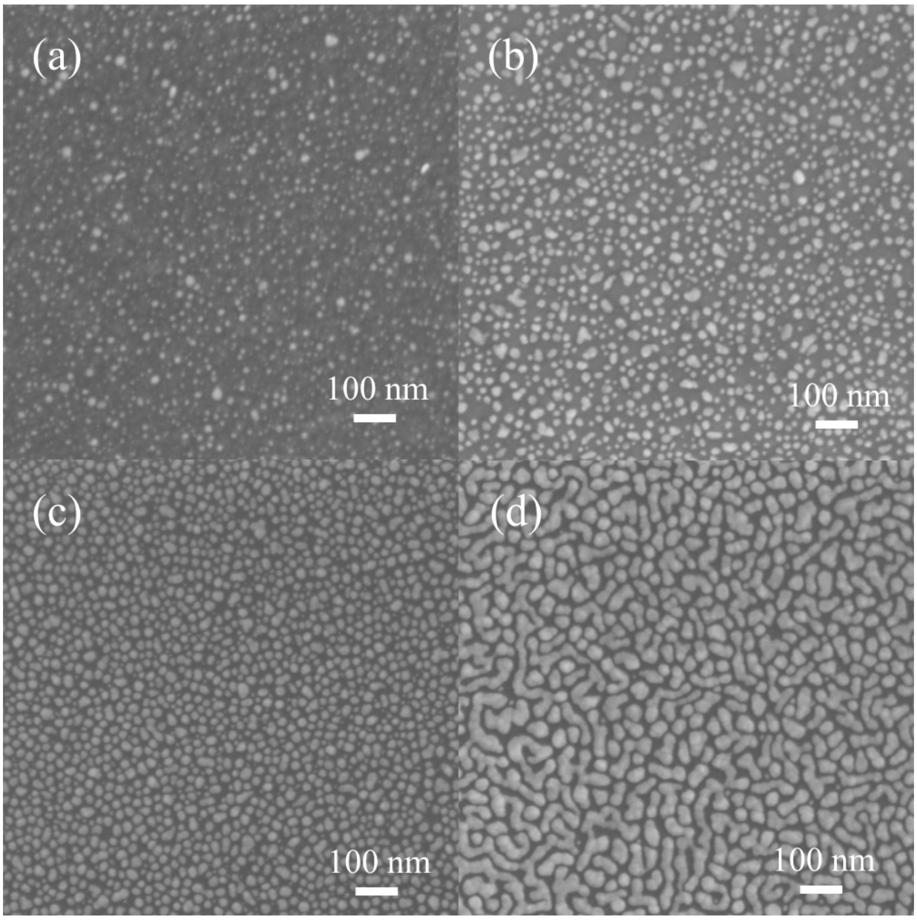

Figure S1 FESEM images of the $Ag/TiO_2/Ti$ foam at different Ag deposition time: (a) 10 min, (b) 20 min, (c) 30 min, (d) 40 min.

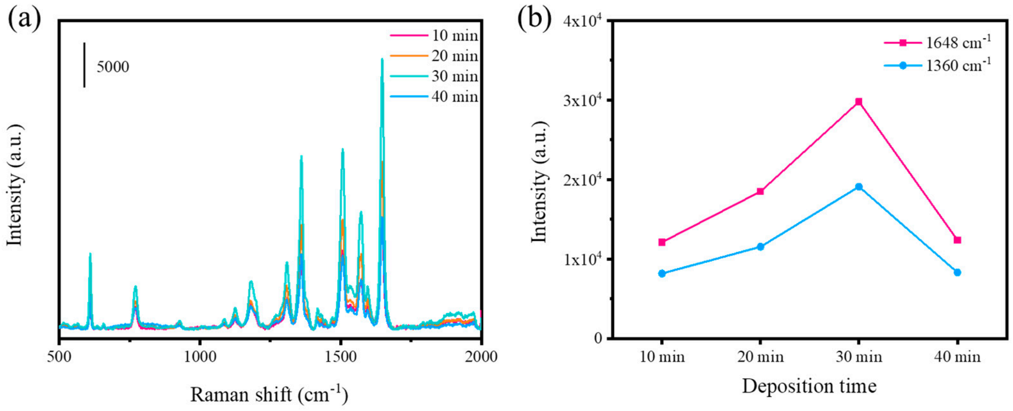

Figure S2 (a) SERS spectra of different Ag deposition time of 10^{-6} M R6G adsorbed on Ag/TiO_2/Ti foams. (b) Corresponding line graphs of SERS intensities of 1648 and 1360 cm^{-1} from 10^{-6} M R6G versus the Ag deposition time.

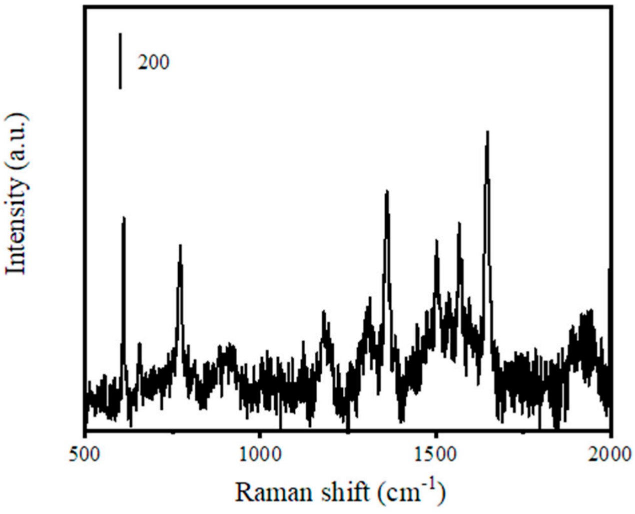

Figure S3 Raman spectra of 10^{-2} M of R6G adsorbed on Ti foil.

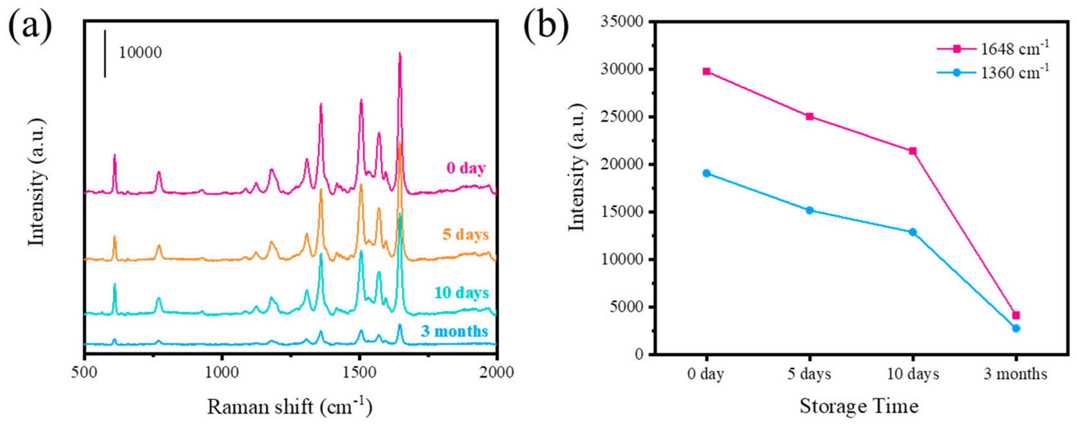

Figure S4 (a)The SERS spectra of 10^{-6} M R6G adsorbed on $Ag/TiO_2/Ti$ foam stored in air for different days. (b) The relationship between storage time of $Ag/TiO_2/Ti$ foam and SERS intensity of R6G at 1648 cm^{-1} and 1360 cm^{-1}.

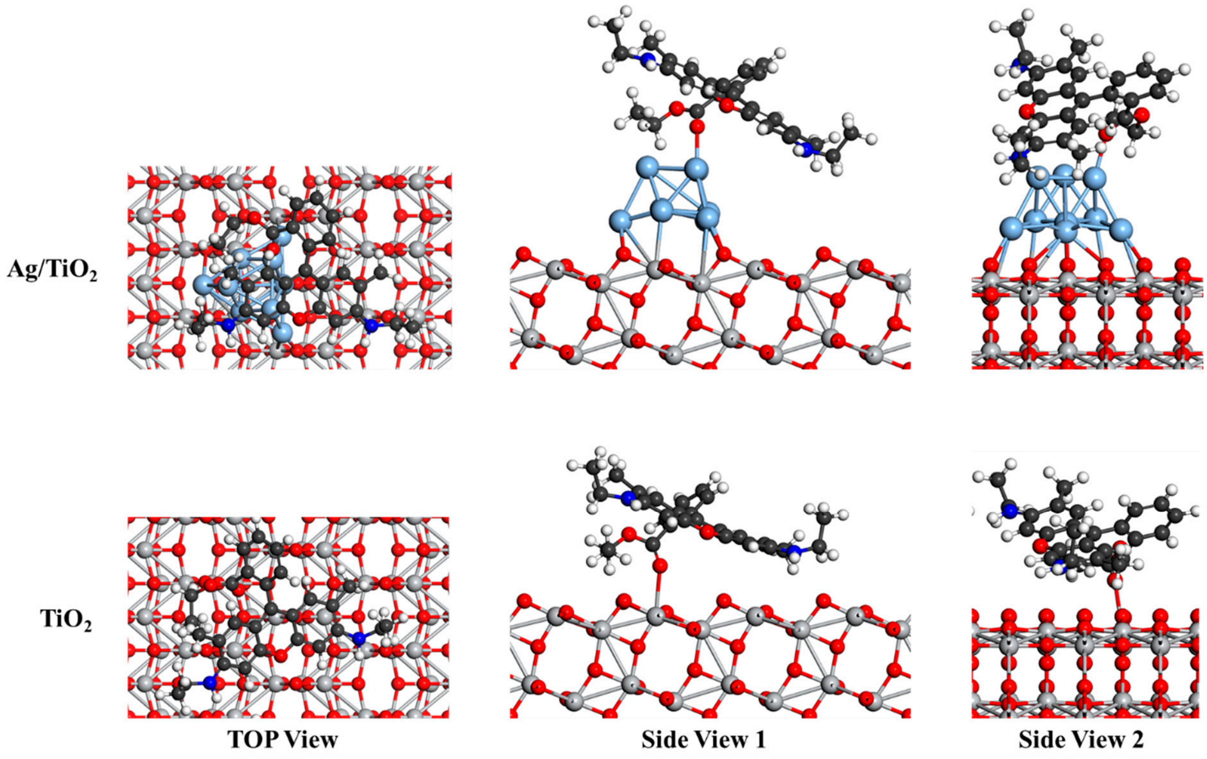

Figure S5 Optimized geometry of R6G on Ag/TiO$_2$ and TiO$_2$.

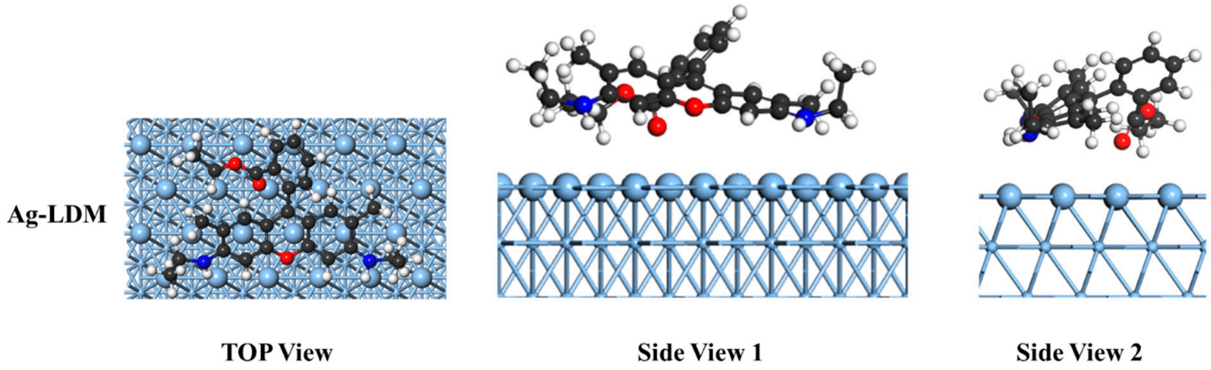

Figure S6 Optimized geometry of R6G on Ag.

Table S1 Elemental composition as obtained from EDS data.

Element	Mass percentage	Atomic percentage
O K	16.19	37.05
Ti K	81.19	62.06
Ag L	2.62	0.89
Total	100.00	

[1] Kresse, G.;Hafner, J. Ab initio molecular dynamics for liquid metals. *Phys. Rev. B* **1993**, *47*, 558-61.

[2] Kresse, G.;Hafner, J. Ab initio molecular-dynamics simulation of the liquid-metal--amorphous-semiconductor transition in germanium. *Phys. Rev. B* **1994**, *49*, 14251-69.

[3] Blöchl, P.E. Projector augmented-wave method. *Phys. Rev. B* **1994**, *50*, 17953-79.

[4] Kresse, G.;Joubert, D. From ultrasoft pseudopotentials to the projector augmented-wave method. *Phys. Rev. B* **1999**, *59*, 1758-75.

[5] Perdew, J.P.;Burke, K.;Ernzerhof, M. Generalized Gradient Approximation Made Simple. *Phys. Rev. Lett.* **1996**, *77*, 3865-8.

[6] Monkhorst, H.J.;Pack, J.D. Special points for Brillouin-zone integrations. *Phys. Rev. B* **1976**, *13*, 5188-92.
